# Influence of Soil Amendment Application on Growth and Yield of *Hedysarum scoparium* Fisch. et Mey and *Avena sativa* L. Under Saline Conditions in Dry-Land Regions

**DOI:** 10.3390/plants14060855

**Published:** 2025-03-09

**Authors:** Ahmad Azeem, Wenxuan Mai, Bilquees Gul, Aysha Rasheed

**Affiliations:** 1 State Key Laboratory of Desert and Oasis Ecology, Xinjiang Institute of Ecology and Geography, Chinese Academy of Sciences, Urumqi 830011, China; ahmadazeem631@yahoo.com; 2 Dr. Muhammad Ajmal Khan Institute of Sustainable Halophyte Utilization, University of Karachi, Karachi 75270, Pakistan

**Keywords:** dry land, soil amendments, saline irrigation, halophytes, growth responses, salt-tolerant plants

## Abstract

Globally, salt stress is one of the most significant abiotic stresses limiting crop production in dry-land regions. Nowadays, growing crops in dry-land regions under saline irrigation is the main focus. Soil amendment with organic materials has shown the potential to mitigate the adverse effects of salinity on plants. This study aimed to examine the ameliorative impact of soil amendment (manure + sandy, compost + sandy, clay + sandy and sandy soil) on the growth, yield, physiological, and biochemical attributes of *Hedysarum scoparium* Fisch. et Mey (HS) and *Avena sativa* L. (OT) under fresh and saline water irrigation in dry-land regions. The results showed that salt stress negatively affected both plant species’ growth, physiological traits, yield, and chloride ions. In response to saline irrigation, plants of both species increased catalase (CAT) and ascorbate peroxidase (APX) activities as part of a self-defense mechanism to minimize damage. Salt stress also significantly raised levels of hydrogen peroxide (H_2_O_2_), malondialdehyde (MDA), and chloride ions (Cl). However, soil amendment treatments like manure + sandy and compost + sandy soil countered the negative effects of saline irrigation, significantly improving plant growth and yield compared with sandy soil. Thus, organic soil amendment is a promising strategy for sustainable crop production under saline irrigation in dry-land regions. This study provides valuable insights into enhancing agricultural production by fostering resilient halophytes and salt-tolerant plant species in challenging environments.

## 1. Introduction

Dry-land regions experience extreme climatic conditions, including high evapotranspiration, limited rainfall, saline water intrusion, and elevated temperatures, which pose significant challenges to agriculture and vegetation growth [1]. In dry-land regions, agriculture is the primary livelihood for the local population. Farmers rely on seasonal rains to cultivate short-duration crops and fodder for livestock [2]. However, irregular rainfall and temperature fluctuations often force them to use saline water and degraded soil, further complicating farming [3,4]. Enhancing soil properties, implementing smart irrigation techniques, and introducing high-yield, salt-tolerant plant species can help improve agricultural productivity in the dry-land regions [5,6,7]. Effective strategies to mitigate salt stress in dry-lands include organic and inorganic fertilization, seed priming, inter-cropping, exogenous phytohormone application, and soil amendments, as well as cultivating salt-tolerant and halophytic plants [8]. Salt-tolerant plant species and halophytic plants can thrive in environments with salinity levels exceeding 150 mM NaCl, making them well suited for regions with high salinity and poor soil quality [9,10]. In addition, they enhance soil fertility by removing excess salts and other toxic substances from the surface [11].

Plants in dry-land regions face various abiotic stresses, including temperature fluctuations, salinity, drought, erosion, and UV radiation [1]. Among these, soil salinity poses the most significant threat to agriculture, water resources, and land productivity, particularly in coastal and dry-land areas worldwide [3,4,5]. Salinity hinders plant growth by inducing water stress and cytotoxicity due to the excessive accumulation of sodium and chloride ions [4].

Additionally, salinity triggers oxidative stress, negatively impacting plant morpho-physiological traits, such as reduced plant height and leaf area [6]. It also lowers chlorophyll a (Chl.a) and b (Chl.b) levels, along with relative water content (RWC) [10]. Salt stress further increases ion toxicity, disrupting nutrient uptake, particularly potassium [8]. High salinity reduces the effective photochemical quantum yield (Φ_PSII_), limiting nitrogen and potassium absorption [12]. Moreover, elevated salt concentrations lead to the accumulation of reactive oxygen species such as H_2_O_2_ and O_2_, causing protein oxidation and lipid peroxidation and further intensifying salinity stress [13,14]. To overcome these salinity effects on plant growth, soil amendment and utilizing saline water in a proper way play important roles in dry-land regions.

Improving soil properties in dry-land areas is crucial for enhancing plant growth while reducing water and fertilizer usage [15]. Soil amendment has proven to be an effective strategy for increasing crop yield and resilience to abiotic stresses under saline irrigation [16,17]. Various materials, such as biochar, gypsum, organic matter, and chemical additives, have been used to enhance soil quality in dry-land regions [18,19]. However, biochar, gypsum, and chemical treatments are not eco-friendly or cost-effective and require highly skilled labor, increasing initial expenses. In contrast, natural manure, organic compost, and clay soil offer sustainable and cost-effective alternatives for improving soil fertility and supporting plant growth in arid conditions [20,21]. Despite their potential, limited research has explored the use of these natural amendments for enhancing plant growth under saline irrigation [22,23].

Integrating salt tolerant plant species along with halophytic plants and soil amendments presents a promising approach for resource-limited farmers to utilize dry-land areas effectively. Several halophyte species, including *Suaeda maritima* (L.) Dumort., *Salicornia europaea* L., *Salsola soda* L., and *Tetraena qatarensis* (Hadidi) Beier & Thulin, have been studied in dry-land regions under saline irrigation [12,13,14]. However, research on the response of *Hedysarum scoparium* Fisch. et Mey. (HS) and Oat (*Avena sativa* L.) in such extreme conditions remains limited. This method offers a sustainable solution for rehabilitating degraded soils and mitigating salt stress in arid regions. Oat (*Avena sativa* L.) (OT) is an annual crop belonging to the Poaceae family [24]. It is salt-tolerant and cultivated worldwide, primarily in the northern hemisphere’s temperate regions [25,26]. *Hedysarum scoparium* Fisch. et Mey. (HS) is an important halophytic shrub species widely distributed in dry-land regions [27,28]. It has the ability to survival under harsh environmental conditions, and the production rate is higher [29,30].

This study examines the effects of soil amendments on the growth and yield of *Avena sativa* L. (OT) and *Hedysarum scoparium* (HS) in dry-land regions under saline irrigation. It also explores oxidative stress and antioxidant enzyme activity in both species. Additionally, the study investigates how soil amendments influence photosynthetic pigment levels in these plants under saline irrigation in an arid condition. This study hypothesizes that soil amendments enhance the growth and productivity of *Hedysarum scoparium* Fisch. et Mey and *Avena sativa* L. by mitigating the adverse effects of saline irrigation in arid conditions.

## 2. Results

### 2.1. Growth Traits and Yield Response

**Figure 1:**
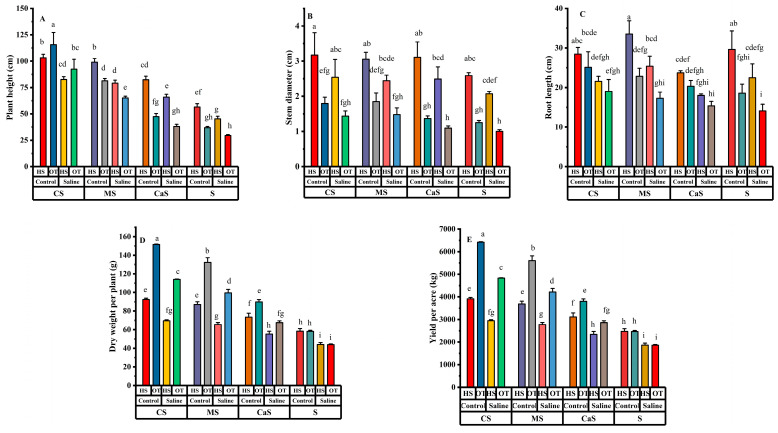
Effects of different irrigation and soil amendment in both plant species: (**A**) plant height, (**B**) stem diameter, (**C**) root length, (**D**) dry weight per plant and (**E**) yield. Error bars above specify the ±SE of three replicates. Different letters indicate the significant difference between parameters. Note: CS = compost + sandy soil, MS = manure + sandy soil, CaS = clay + sandy soil, and S = sandy soil. Control = fresh water irrigation; saline = saline water irrigation. HS = *Hedysarum scoparium* and OT = oat.

A general linear univariate analysis of variance (ANOVA) was used to evaluate the effect of the independent variables, i.e., plant species, water treatments, soil treatments, and their interaction on growth traits and yield. This analysis was conducted with different soil amendment treatments and water treatments for both plant species. The results indicate that the independent factor has significant effects on all growth traits parameters and yield. In the interaction effects, plant height (PH) has a significant difference between the Species × Soil and Water × Soil. PH of both plant species had a significant difference in every soil amend treatment and different types of irrigation water, as shown in Figure 1A. OT has a higher PH in compost + sandy (CS) and manure + sandy (MS) soil treatment as compared with clay + sandy (CaS) and sandy soil (S) within both water treatments. Similarly, on the other hand, HS maintained a good PH under CS, MS, and CaS soil treatments compared with S soil within both water treatments, as shown in Figure 1A. In all interaction effects, stem diameter (SD) was non-significant. Both plant species’ SD significantly changed in all soil amendment treatments, as shown in Figure 1B. OT has a higher SD in CS and MS soil treatments as compared with CaS and S soil within both water treatments. Similarly, on the other hand, HS maintained a good SD under CS, MS, and CaS soil treatments compared with S soil within both water treatments as shown in Figure 1B. In the interaction effects, RL only have significant results between Species × Soil, and the rest of the interaction effects were non-significant.

RL of both plant species decreased under saline irrigation as compared with fresh water irrigation in all soil amendment treatments, as shown in Figure 1C. The rate of RL reduction in both plants was lower in all soil amendment treatments than the S soil under saline irrigation. Root length plays a crucial role in plant adaptation to saline irrigation, as it directly affects water and nutrient uptake. However, salinity stress often leads to a significant reduction in root length due to osmotic stress, ion toxicity, and oxidative damage that was found in both plant species. Shorter roots limit the plant’s ability to access water and essential nutrients, exacerbating growth inhibition and reducing overall yield. At the same time, soil amendments help to enhance the soil conditions that can assist plants to improve root development under saline environments. DW has a significant effect on all interaction combinations, DW of both plant species was decreased in saline irrigation as compared with freshwater irrigation in all soil amendment treatments. Meanwhile, the DW of both plant species at CS, MS, and CaS was higher than the S soil under both irrigation treatments, as shown in Figure 1D. Yield has significant effects in all interaction combinations. The yield of both plant species decreased in saline irrigation as compared with freshwater irrigation in all soil amendment treatments. The yield of both plant species at CS, MS and CaS was higher as compared with S soil under both irrigation treatments as shown in Figure 1E.

### 2.2. Chlorophyll Content and Fluorescence Parameters Under Soil Amendments

**Figure 2:**
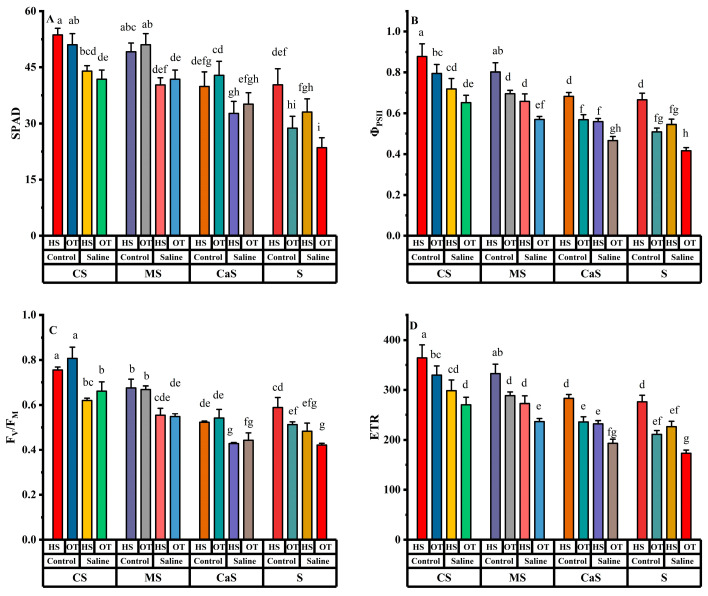
Effect of different irrigation and soil amendment in both plant species; (**A**) chlorophyll content (SPAD), (**B**) quantum yield of photosystem II (Φ_PSII_), (**C**) maximal photochemical efficiency of photosystem II (F_V_/F_M_), and (**D**) electron transport rate (ETR). Error bars above specify the ±SE of three replicates. Different letters indicate the significant difference between parameters. Note: CS = compost + sandy soil, MS = manure + sandy soil, CaS = clay + sandy soil and S = sandy soil. Control = fresh water irrigation; saline = saline water irrigation. HS = *Hedysarum scoparium* and OT = oat.

Variations in chlorophyll content (SPAD), quantum yield of photosystem II (Φ_PSII_), maximal photochemical efficiency of photosystem II (F_V_/F_M_), and electron transport rate (ETR) have been determined in all soil amendment treatments along with different water irrigation as shown in Figure 2. SPAD, Φ_PSII_, F_V_/F_M_ and ETR all have significant effects on all single factors like species, water, and soil. In the interaction effects, only F_V_/F_M_ had a significant effect in all interactions, and SPAD only had a significant effect in the Species × Soil interaction. The rest of the other parameters are non-significant in all interaction effects. The SPAD values in all soil amendment treatments decreased under saline irrigation compared with freshwater irrigation. At the same time, CS and MS soil amendment treatments have higher values of SPAD as compared with the other two soil amendment treatments under both water irrigation, as shown in Figure 2A.

The values of quantum yield of photosystem II (Φ_PSII_), maximal photochemical efficiency of photosystem II (F_V_/F_M_) and electron transport rate (ETR) have been increased in all soil amend treatments under saline irrigation as compared with freshwater irrigation. Under CS and MS soil treatment values of Φ_PSII_, F_V_/F_M_ and ETR were higher in both water treatments as compared with CaS and S treatment, as shown in Figure 2B–D. Chlorophyll content and fluorescence parameters are key indicators of photosynthetic efficiency, particularly under saline irrigation. Salinity stress disrupts chlorophyll synthesis, leading to a decline in photosynthetic activity due to reduced light absorption and impaired electron transport. Lower chlorophyll levels weaken carbon assimilation, ultimately affecting plant growth and yield.

### 2.3. Response to Photosynthesis Pigments Under Soil Amendments

**Figure 3:**
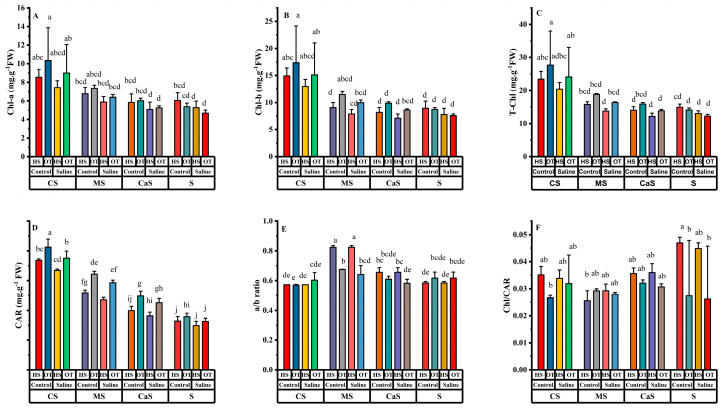
Effect of different irrigation and soil amendments in both plant species. (**A**) Chlorophyll a (Chl-a), (**B**) Chlorophyll b (Chl-b), (**C**) Total chlorophyll (T-Chl), (**D**) Carotenoids (CARs), (**E**) a/b ratio, and (**F**) Chl/CAR. Error bars above specify the ±SE of three replicates. Different letters indicate the significant difference between parameters. Note: CS = compost + sandy soil, MS = manure + sandy soil, CaS = clay + sandy soil and S = sandy soil. Control = fresh water irrigation; saline = saline water irrigation. HS = *Hedysarum scoparium* and OT = oat.

Variations in ChI-a, Chl-b, T-Chl, carotenoid (CAR), a/b ratio, and ChI/CAR have been found in all soil amendment treatments along with different water irrigation as shown in Figure 3. ChI-a has a significant effect on the independent factor, i.e., water and soil. At the same time, the rest of the interaction effects were non-significant. Similarly, Chl-b and T-Chl values were significant in the single factor analysis, except T-Chl at species was non-significant. In the interaction effects, both Chl-b and T-Chl were non-significant. CAR has a significant response under single factor analysis, but the interaction only has significant results at Species × Soil. The a/b ratio has a significant response at species and soil in the single factor analysis, but in the interaction, effects only have a significant result at Species × Soil. At the same time, ChI/CAR have a significant result in single factor analysis only in species, and interaction only has in Species × Soil.

ChI-a values decreased in all soil amendment treatments under saline irrigation compared with freshwater irrigation, but the decrease in Chl-a values was mostly non-significant due to soil amendment effects, as shown in Figure 3A.

Chl-b and T-Chl values in every soil amendment treatment maintained non-significant results within the same soil amendment treatment in both water quality irrigation as shown in Figure 3B,C. In contrast, CS and MS have higher values of Chl-b and T-Chl under both water quality irrigation as compared with the other two soil treatments. CAR value decreased in all soil amend treatments under saline irrigation as compared with freshwater irrigation. The results were significant in all soil amendment treatments within each soil treatment due to different water quality irrigation, as shown in Figure 3D. The a/b ratio in all soil amendment treatments maintains non-significant results under both water quality irrigation, as shown in Figure 3E. Similarly, ChI/CAR mostly maintained non-significant results within the soil treatment under both water quality irrigation, as shown in Figure 3E.

### 2.4. Antioxidant Enzyme Activity and Oxidative Damage Makers Under Soil Amendment

Hydrogen peroxide (H_2_O_2_), malondialdehyde (MDA), protein, ascorbate peroxidase (APX), and catalase (CAT) have significant results in all single factor analyses. The interaction effects H_2_O_2_ and protein have a significant effect only in the Species × Soil. APX has non-significant results in all interaction combinations. Furthermore, MDA has significant results within Species × Water and Species × Soil, but CAT have significant results within Species × Soil and Water × Soil.

**Figure 4:**
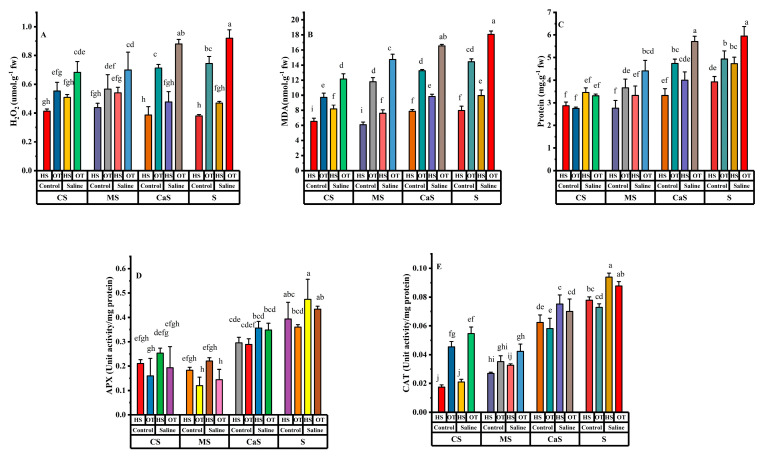
Effect of different irrigation and soil amendments in both plant species. (**A**) Hydrogen peroxide (H_2_O_2_), (**B**) Malondialdehyde (MDA), (**C**) protein, (**D**) ascorbate peroxidase (APX), and (**E**) Catalase (CAT). Error bars above specify the ±SE of three replicates. Different letters indicate the significant difference between parameters. Note: CS = compost + sandy soil, MS = manure + sandy soil, CaS = clay + sandy soil and S = sandy soil. Control = fresh water irrigation; saline = saline water irrigation. HS = *Hedysarum scoparium* and OT = oat.

The values of H_2_O_2_, MDA, protein, APX, and CAT were increased in all soil amend treatments under saline irrigation as compared with fresh water irrigation as shown in Figure 4. The values of all antioxidant enzyme activity were significantly different within the same soil treatment under different water quality irritation. The values of H_2_O_2_, MDA, protein, APX and CAT were higher at S and CaS soil treatments under saline irrigation as compared with CS and MS as shown in Figure 4A–E. Soil amendments, such as CS and MS, can enhance antioxidant defense mechanisms by improving soil structure, nutrient availability, and water retention. This helps mitigate oxidative stress, supporting better physiological performance and yield under saline conditions as shown in Figure 1. Understanding the interaction between antioxidant enzyme activity, oxidative damage, and plant productivity is crucial for developing strategies to enhance crop resilience in saline environments.

### 2.5. Ion’s Concentration

Soil amendments and water treatments have significant variations in the ion concentration in plant leaves in both plant species. Chloride ions (Cl), ammonium ions (NH_4_), and nitrate ions (NO_3_) have significant results in all single factors, but in combination, except chloride ions (Cl) have a non-significant result in species. In the interaction effects, all ion concentrations have significant effects in Species × Soil; the rest of the other combinations have non-significant results.

**Figure 5:**
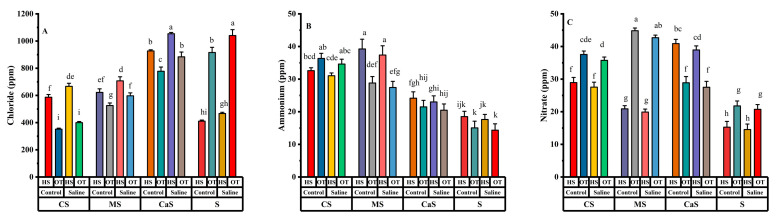
Effects of different irrigation and soil amendment in both plant species. (**A**) Chloride ions (Cl), (**B**) ammonium ions (NH_4_), and (**C**) nitrate ions (NO_3_). Error bars above specify the ±SE of three replicates. Different letters indicate the significant difference between parameters. Note: CS = compost + sandy soil, MS = manure + sandy soil, CaS = clay + sandy soil and S = sandy soil. Control = fresh water irrigation; saline = saline water irrigation. HS = *Hedysarum scoparium* and OT = oat.

Cl concentration has a significant result in all soil amend treatments under both water quality irrigation as shown in Figure 5A. Cl concentration at MS and CS is lesser as compared with S and CaS. Similarly, NH_4_ have significant results in CS and MS soil treatment under both irrigations, but CaS and S have non-significant results as shown in Figure 5B. NH_4_ concentration is higher at CS and MS treatment under both water irrigation as compared with CaS and S treatments. NO_3_ has a significant result within soil treatment in every soil amend treatment under both water irrigation as shown in Figure 5C. NO_3_ concentration is higher at CS, MS and CaS treatments under both water irrigation as compared with the S soil.

### 2.6. Correlation Traits

**Figure 6:**
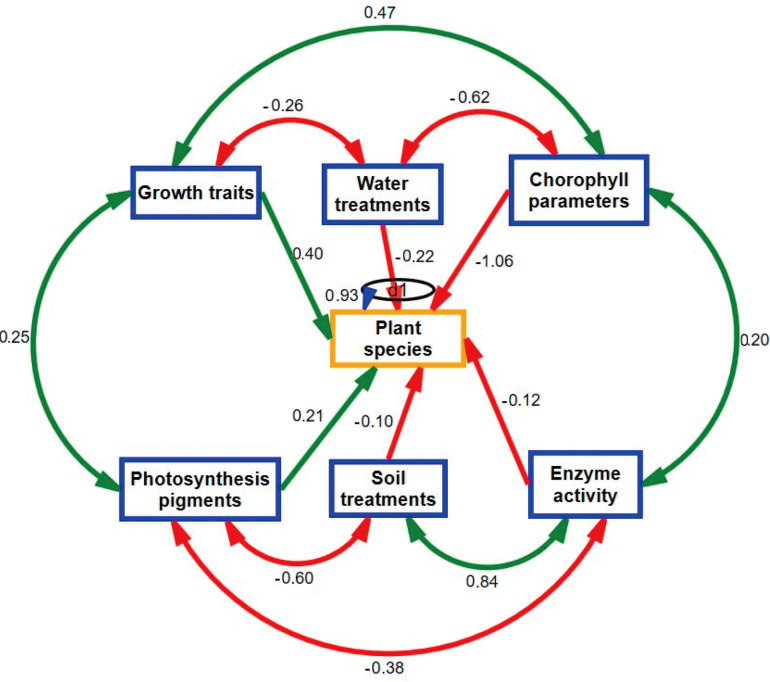
Structural model equation relating growth traits, chlorophyll parameters, photosynthesis pigment and enzyme activity of two plant species among water quality treatments, and soil amendments. Green lines indicate positive relationship between growth traits, chlorophyll parameters, photosynthesis pigment and enzyme activity. The red lines indicate the negative relationship between growth traits, chlorophyll parameters, photosynthesis pigment and enzyme activity among water treatments, soil amendments, and plant species.

The Pearson correlational coefficient was performed to check the relationship between growth traits parameters, photosynthesis pigment, chlorophyll parameters, enzyme activity, water treatments, soil amendment treatments, plant species, and average values of experimental data, as shown in Figure S1. Water treatments, plant species, and soil amendment treatments had a negative relationship with all growth traits, photosynthesis pigment and chlorophyll parameters. On the other hand, enzyme activity had a positive relationship with water treatments, plant species, and soil amendment treatments, as shown in Figure S1. The structural equation modeling was well fitted to the average data of the experiment (Chi-square = 127.7, *p* = 0.000, df = 7, CFI = 0.640, RMSE = 0.003), as shown in Figure 6. Water treatment has a negative relationship with growth traits, plant species and chlorophyll parameters. Similarly, soil treatment has a negative relationship with plant species and photosynthesis pigment but a positive relationship with enzyme activity, as shown in Figure 6.

## 3. Discussion

Saline water irrigation and poor soil quality are major challenges in dry-land regions all over the world [9]. Studies have shown that poor soil quality and saline irrigation primarily impact early growth stages, as well as key physiological and biochemical processes, ultimately reducing crop yields in these regions [31,32]. The utilization of soil amendment techniques along with salt-tolerant plant species is the only eco-friendly approach to cope with these challenges in dry-land regions. This study assessed salt stress effects on various parameters of HS and OT with the application of soil amendments. Saline irrigation reduced all growth traits, including plant height (PH), stem diameter (SD), root length (RL), dry weight per plant (DW), and, ultimately, yield in both plant species. However, the application of soil amendments improved the growth traits compared to sandy soil under both fresh and saline irrigation. Several researchers have reported similar findings, showing that all growth parameters decline under salt stress but improve in treated soil [17,33]. The reduction in growth attributes may be due to the accumulation of various osmolytes, restricted cell division, and changes in the activity of metabolic enzymes and pathways, all of which play a pivotal role during salt stress [34]. Under saline irrigation conditions, both plant species exhibited a decline in key photosynthetic parameters, including chlorophyll content (SPAD), quantum yield of photosystem II (Φ_PSII_), maximal photochemical efficiency of photosystem II (F_V_/F_M_), and electron transport rate (ETR). This reduction may be attributed to enhanced chlorophyllase enzyme activity and increased reactive oxygen species (ROS) production [35]. Additionally, it resulted in decreased nitrogen uptake, inhibition of photosynthesis, and instability in the protein assembly of photosynthetic pigments [36,37]. Soil amendments helped plants to alleviate the negative effects of salinity, supporting plant growth and maintaining a more balanced physiological state. These amendments likely improved soil structure, enhanced nutrient availability, and helped regulate ion homeostasis, thereby mitigating salt-induced stress in plants [15]. In this study, soil amendments like CS and MS treatments increased SPAD, Φ_PSII_, F_V_/F_M_ and ETR in both plant species. Furthermore, these soil amendment treatments also increased photosynthesis pigments like T-ChI, CAR, a/b ratio and ChI/CAR in both plant species. The application of soil amendments in combination with salts resulted in increased chlorophyll and CAR content compared to salt-stressed plants, as previously observed in oat, *Melilotus officinalis* (L.) Pall, tomato, *Atriplex canescens* (Pursh) Nutt and summer savory [38,39]. An increase in nitrogen uptake and a decrease in chlorophyllase enzyme activity may explain the reduction in chlorophyll degradation [40]. The present results showed that MDA and H_2_O_2_ levels increased with higher salinity levels compared to freshwater irrigation in both plant species across all soil amendment treatments. Elevated MDA and H_2_O_2_ levels indicate oxidative stress, which affects several vital cellular functions in plants by causing protein, DNA, and RNA oxidation, as well as lipid peroxidation [41]. MDA is a byproduct of lipid peroxidation, a process where reactive oxygen species (ROS) damage cell membrane lipids, leading to membrane instability and impaired cellular function [25]. High levels of MDA indicate increased oxidative damage, which can compromise cell integrity and disrupt normal metabolic activities. Similarly, H_2_O_2_ is a type of ROS that plays a dual role in plant cells. It acts as a signaling molecule at low concentrations but becomes toxic at higher levels, leading to oxidative damage to proteins, nucleic acids (DNA and RNA), and lipids [26]. The accumulation of H_2_O_2_ under saline conditions further exacerbates stress by impairing photosynthesis and enzymatic functions [42]. However, soil amendments like MS and CS alleviated salt stress conditions and reduced MDA and H_2_O_2_ levels in the leaves of both plant species. Similar findings on the effects of soil amendments under abiotic stress have been reported in previous studies on bean seedlings and wheat [43,44].

Salinity-induced oxidative stress impairs plant growth and development due to the accumulation of reactive oxygen species (ROS), which can be toxic to plants under stress conditions [44]. In plants, ROS are neutralized by key antioxidant enzymes, including APX, and CAT [45]. Phytohormones, such as abscisic acid (ABA), salicylic acid (SA), and jasmonic acid (JA), interact with these antioxidant enzymes to enhance stress tolerance [46]. ABA, a key hormone in drought and salt stress responses, regulates CAT and APX activity to maintain cellular redox balance [47]. Similarly, SA modulates APX activity, improving the plant’s ability to detoxify ROS and sustain photosynthetic efficiency under saline conditions [48]. However, under extreme stress, plants often struggle to detoxify ROS adequately, leading to oxidative stress. The utilization of organic materials like manure and compost as soil amendments, further reduced enzymatic activity including APX and CAT compared with sandy soil treatment plants. This reduction suggests that oxidative stress may have occurred due to damage in the antioxidant defense system [49].

The present study showed that chloride ions (Cl) increased under salt stress but decreased with MS and CS soil treatments as compared with the other two soil treatments, both in saline and freshwater conditions. These findings align with previous research in wheat, tomato and maize, which reported reduced Cl uptake in plants under organic treated soil, potentially due to the high adsorption capacity of manure and compost that alleviates osmotic stress on plants [5,47]. Additionally, manure and compost may enhance mineral nutrient availability and improve soil chemical and physical properties, further reducing Cl uptake. In contrast, ammonium (NH_4_) and nitrate (NO_3_) ions, which decreased under saline conditions, showed an increased with manure and compost soil amend application in both saline and fresh water irrigation, similar to findings in bean plants [47,50].

Salt stress poses a significant challenge for agricultural lands worldwide in dry-land regions. Salt stress impacts various growth parameters, including morphological, physiological, biochemical, yield, and mineral ion composition in plants [51]. Previous studies have shown that salt stress mainly affects early growth stages and disrupts physiological and biochemical processes, leading to reduced yields in many crops in dry-land regions [3,23,49]. This study aimed to assess the impact of salinity stress on several parameters in HS and OT under dry-land conditions. Both the plant species showed a different trend under these harsh environmental conditions because both plant species have different adaptation styles under saline conditions. HS showing a better growth response in all soil amendment treatments, but OT show better growth only in MS and CS under saline irrigation. Under saline irrigation, OT and HS exhibit distinct physiological responses to stress. OT demonstrates a stronger antioxidative defense system, with higher activity of key enzymes such as APX and CAT, enabling more efficient ROS detoxification and membrane stability [26]. In contrast, HS experiences greater oxidative stress, reflected in elevated levels of MDA and H_2_O_2_, leading to increased lipid peroxidation and reduced photosynthetic efficiency [29]. This divergence suggests that OT has a more adaptive mechanism for maintaining chlorophyll content and overall growth under salt stress, whereas *Hedysarum scoparium* is more vulnerable to oxidative damage [2,22].

Organic amendments like manure and compost, improve soil pH by buffering excessive salinity, creating a more balanced environment for root development and nutrient uptake. They also increase organic matter content, which enhances soil fertility by providing essential nutrients such as nitrogen, phosphorus, and potassium, crucial for plant metabolism [52]. Furthermore, manure and compost improve soil texture and structure, increasing water retention and reducing leaching in sandy soils, ensuring a steady supply of moisture and nutrients to plant roots [13,53]. This enhanced soil environment supports root expansion, boosts microbial activity, and strengthens the plant’s ability to withstand salt-induced stress, ultimately leading to healthier growth and improved yield [54]. These soil amendments like manure and compost are highly scalable in dry-land farming, particularly in regions facing salinity and poor soil fertility. At the same time, their effectiveness depends on factors such as availability, transportation costs, and integration into existing farming practices [38,55]. In large-scale farming, compost and manure can be applied through mechanized spreading, and their benefits, such as improved soil moisture retention and nutrient availability, make them valuable for long term productivity [56]. However, scalability challenges arise in sourcing sufficient organic material, especially in areas with limited livestock or composting infrastructure. While manure and compost improve soil structure and fertility, excessive or unbalanced use can cause nutrient imbalances, soil acidification, and microbial competition for nitrogen. Improper management may also lead to salt accumulation if organic materials contain residual salts. Large-scale adoption depends on a steady and affordable supply of organic matter, which can be limited in arid regions. Over-reliance on livestock waste or composted residues may become unsustainable if demand exceeds supply, increasing costs and environmental strain. Additionally, organic amendments require more labor for collection, processing, and application than synthetic fertilizers, making them less feasible for resource-limited farmers without subsidies or local composting programs.

### Purposed Mechanism of Soil Amendment to Cope with Salt Stress

The application of soil amendment has been shown to effectively increase plant growth rates and yield across a range of salinity levels. Studies have found that adding manure and compost as soil amendment materials enhances all measured vegetative characteristics at various salt concentrations compared to plants without soil amendment treatments. Soil amendment with organic materials contributes to improved soil structure, water retention, and nutrient availability, collectively strengthening plant resilience to saline conditions [57,58]. Additionally, soil amendment with organic materials application significantly reduces sodium uptake while enhancing potassium uptake, which helps maintain a healthier ionic balance in plants subjected to salt stress [59].

Soil amendments lead to a reduction in soluble sugar levels under stress conditions. Sugars are essential in maintaining cellular membrane integrity and play a vital role in the oxidative stress response by scavenging reactive oxygen species in saline environments [60]. Under stress, elevated sugar levels arise from the breakdown of larger carbohydrate molecules, aiding in cell turgidity and protecting membranes and other cellular structures from adverse effects. Soil amendment with organic materials reduces MDA and H_2_O_2_ production, which strengthens cellular membranes and lowers electrolyte leakage [61].

Chlorophyll concentration, closely linked to photosynthesis, has been found to increase significantly with soil amendment application. Additionally, organic soil amendment modulates the expression of stress-responsive genes, as studies have shown it can up-regulate genes involved in antioxidant defense, osmotic balance, and ion transport [62]. This genetic regulation aids plants in coping with oxidative and ionic stresses associated with saline environments [63]. In summary, organic soil amendment effectively reduces the adverse effects of salt stress in plants, enhances membrane stability, nutrient uptake, chlorophyll synthesis, and regulates stress-responsive genes in dry-land regions [64]. Overall, organic soil amendment considerably improves plant resilience and productivity under saline conditions.

For farmers and policymakers in dry-land regions, integrating soil amendments such as manure, compost, biochar, and gypsum can significantly enhance soil fertility, water retention, and crop resilience under saline conditions [65]. A balanced amendment strategy is essential, combining organic materials like compost and manure to increase soil organic matter with mineral-based amendments like gypsum to reduce salinity and improve soil structure [66]. Policymakers can support this by investing in composting facilities, providing subsidies for organic amendments, and promoting training programs on sustainable soil management [67]. However, using compost and manure in sandy dry-land soils has limitations, including rapid decomposition, nutrient leaching, and labor-intensive management, which reduce their long-term effectiveness in improving soil fertility [68]. Additionally, challenges such as limited availability, high transportation costs, and the risk of introducing pathogens or weed seeds make large-scale application difficult for resource-constrained farmers. Future research should focus on developing slow-release organic amendments, microbial inoculation, and alternative organic inputs to enhance nutrient retention and soil resilience. Moreover, integrating compost, manure, biochar, and mineral amendments with policy-driven economic models for cost-effective distribution can improve soil health and ensure long-term agricultural sustainability in dry-land regions.

## 4. Materials and Methods

A field experiment was conducted at the Sindh Engro Coal Mining Company’s experimental site in Tharparkar, Block II, Islamkot, Sindh province, Pakistan. The study spanned from 10 October 2023, to 13 March 2024, for OT, and from 10 November 2023 to 13 March 2024 for HS. The site is characterized by extreme weather, with long, hot summers where temperatures can soar up to 48 °C, and short winters with lows around 8 °C. The region receives annual rainfall of about 100 mm, but the evaporation rate is considerably higher, at 2600 mm per year. The soil is predominantly dry and sandy, with minimal nutrient content. Monthly temperature and humidity data of the experimental period are shown in Figure S2.

### 4.1. Experimental Design

**Figure 7:**
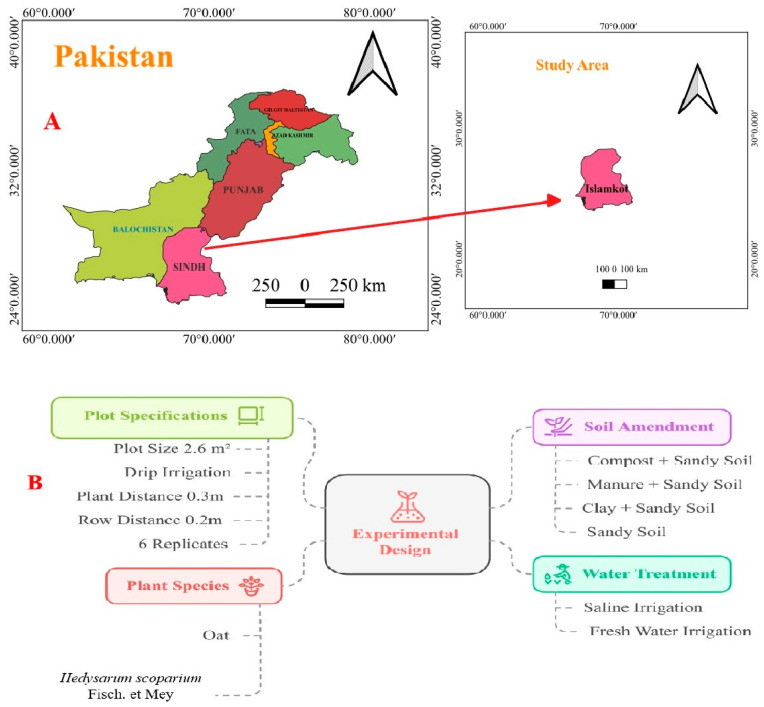
(**A**) Location of the study area and (**B**) experimental design.

Three types of soil amendments, natural manure, organic compost, and clay, were tested with sandy soil using a completely randomized block design. The amendments were mixed with the sandy soil in 2.6 m^2^ plots at a rate of 5.7 kg per plot. Each soil treatment was replicated six times, and an additional six plots with untreated sandy soil were used as control plots. The seeds of these plant species were imported from China to Pakistan. For this study, we selected healthy seeds of uniform size to ensure consistency across treatments. These seeds were planted in the experimental field according to the randomized block design, with six replicates per soil amendment treatment. Plant spacing was set at 0.3 m between plants and 0.2 m between rows within each plot. Figure 7 shows the study area’s location and experimental design [22]. Table S1 describes the amended soil’s physical and chemical properties.

Before sowing, the field was prepared by applying urea fertilizer at a 240 kg/ha as previous studies recommended [23]. Fresh water was applied for the first 20 days during the germination and seedling establishment phases. To assess the effects of saline water irrigation on the growth of HS and OT under different soil amendment treatments, irrigation with saline water began 20 days after sowing of both plant species, i.e., saline irrigation started on 1 November for OT and saline irrigation started on 31 December for HS. One set of plots, representing the four soil amendment treatments with three replicates each, was irrigated with saline water. A parallel set with three replicates per treatment was irrigated with fresh water, serving as the control. The physical and chemical properties of both fresh and saline water used in the experiment are detailed in Table S2 [49].

A drip irrigation system was employed, featuring a water meter on the main irrigation line to regulate the frequency of water and fertilizer applications. Each emitter in the drip line was calibrated to release water at a rate of 3 L/h. The irrigation system operated for 15 min per cycle during both saline and freshwater irrigation, providing a consistent watering rate of 6 mm/day to the field. The watering was performed every day.

### 4.2. Chlorophyll Content and Chlorophyll Fluorescence Parameters

At the end of the experiment, on 13 March 2024, leaf chlorophyll content (SPAD) was measured in the leaves of both plant species in every treatment with three replicates using a portable chlorophyll meter (SPAD; Oakoch OK-Y104,Shenzhen China). A PAM fluorometer 2500 (Heinz Walz GmbH, Effeltrich, Germany) was used to measure chlorophyll fluorescence parameters of both plant species. Chlorophyll fluorescence was estimated on fully developed leaves using a pulse-modulated fluorometer (PAM 2500, Walz, Germany). The leaf was dark-adapted for 25 min using leaf clips. The minimal fluorescence (F_o_) value was measured after applying light flash (<0.1 µmol photon m^−2^ s^−1^) on the dark-adapted leaf, while the maximal fluorescence (F_M_) value was obtained by imposing a saturating pulse (10,000 µmol photons m^−2^ s^−1^) for 0.6 s. The F_o_ and F_M_ values were used to calculate the maximum photochemical quantum yield(Φ_PSII_) (F_V_/F_M_ = F_M_ − F_o_/F_M_). The electron transport rate (ETR) was calculated according to the method of Krall and Edwards [69].

### 4.3. Photosynthesis Pigments

Fresh leaves of both plant species in three replicates per treatment were homogenized in 80% acetone using chilled mortar and pestle. The homogenate was centrifuged at 3000× *g* and the supernatant was collected to measure absorbance at 663.2, 646.8 and 470 nm (Beckman-Coulter DU-730, UV–vis spectrophotometer, Boston, USA). Contents of chlorophyll a, b, total chlorophyll and total carotenoid were calculated with equations suggested by Lichtenthaler [70].

### 4.4. Hydrogen per Oxide (H_2_O_2_) and Malondialdehyde (MDA) Content

Fresh leaves of both plant species in three replicates per treatment were homogenized in 5 mL of 3% Tri-chloroacetic acid (TCA), followed by centrifugation at 12,000× *g* for 15 min at 4 °C to prepare extracts. Accumulation of H_2_O_2_ in leaf tissues was estimated according to the KI reagent assay of Loreto and Velikova, [71]. The extent of lipid peroxidation was estimated by determining the accumulation of MDA. MDA contents were determined by using TCA/TBA reagent according to Heath and Packer [72].

### 4.5. Antioxidant Enzymes Activities

Extraction of antioxidant enzymes was performed by following the protocol of Hameed et al. [73]. Catalase (CAT, EC 1.11.1.6) activity was assayed via Abey’s [74] method at 240 nm. Ascorbate peroxidase (APX, EC 1.11.1.11) activity was examined according to Nakano and Asada, [42] at 290 nm. Protein contents of enzyme extracts were determined according to Bradford’s protocol method [75], and enzyme activities were expressed as units per milligram of the protein. All antioxidant enzymes activities were determined by using the plant leaves of three replicates of every treatment of both the plant species.

### 4.6. Growth Traits

The plants of both plant species were harvested. The height and root length of both plant species in each replicate, under different water treatments and soil amendments, were measured using a measuring tape. Shoot fresh weight and root fresh weight were recorded using a weighing balance. These fresh weight measurements were used to calculate the yield by multiplying the total fresh weight per plant by the plant density (28,800 plants per acre) to determine the yield per acre. Following the fresh weight measurements, all plants were placed in an oven for 40 h at 72 °C. After drying, the dry weight was recorded using a weighing balance to determine the biomass per plant [76,77].

### 4.7. Ions Analysis

To determine chloride (Cl), ammonium (NH_4_), and nitrate (NO_3_) concentrations, water-based extracts were prepared (Shoukat et al.) [31]. Cl^−^, NO_3_ and NH_4_ were analyzed by Ion Selective Electrode along with the BANTE instrument. All ion analyses were determined by using the plant leaves of three replicates of every treatment of both the plant species.

### 4.8. Statistical Analysis

A general linear univariate analysis of variance (ANOVA) was used to assess the effects of independent variables such as plant species, water treatments, soil amendments, and their interactions on growth traits, chlorophyll fluorescence parameters, photosynthetic pigments, oxidative stress, antioxidant enzyme activities, and ions analyses. The statistical analysis was performed using SPSS 22. Interaction graphs were generated using Tukey’s test (*p* < 0.05) with Origin Pro 2024b. Structural modeling equation was conducted by using Amos SPSS and correlation analyses were also conducted using Origin Pro 2024b to explore relationships among growth traits, photosynthetic pigments, chlorophyll fluorescence parameters, enzyme activities, oxidative stress, and ion concentrations. All figures were created using Origin Pro 2024b for clear and effective data visualization.

## 5. Conclusions

This research indicates that soil amendment application mitigates the adverse effects of saline irrigation on the growth of HS and OT plants under dry-land conditions. Saline irrigation typically reduces yield, growth (including PH, SD, and DW), physiological parameters (photosynthesis pigment, chlorophyll content, and carotenoid content), increases enzyme activities and chloride ions concentration in both plant species. Organic soil amendment helps alleviate these adverse impacts by enhancing chlorophyll content, yield, and growth parameters under saline conditions of both plant species. This improvement occurs through a reduction in antioxidant activity, H_2_O_2_, and MDA in organic soil amendment-treated plants. Organic soil amendments (manure and compost) are particularly effective in boosting the yield and growth of both plant species under saline irrigation in dry-land regions.

## Data Availability

Data will be available upon demand.

## Supplementary Materials

The following supporting information can be downloaded at https://www.mdpi.com/article/10.3390/plants14060855/s1. Figure S1. Correlation analysis between plant species, water treatment, soil amendment, growth traits, Chlorophyll parameters, photosynthesis pigments and enzyme activity; Figure S2. Average monthly temperature and humidity of the experimental period; Table S1. Physical and chemical properties of amend soil treatments; Table S2. Water Quality parameters.
