# Supplementary material for: Influence of Soil Amendment Application on Growth and Yield of Hedysarum scoparium Fisch. et Mey and Avena sativa L. Under Saline Conditions in Dry-Land Regions
Source: Plants (Basel). 2025 Mar 9;14(6):855. doi: 10.3390/plants14060855 (PMC11945427; doi:10.3390/plants14060855)
Supplement: Supplementary file 1 [file plants-14-00855-s001.zip › plants-3491627-supplementary.pdf]

Table S1. Physical and chemical properties of amend soil treatments

Soil Treatments	Texture Class	Moisture (%)	pH	ECe(2:5) (dS/cm)	soil salinity (ppm)	WHC (%)	Cl (ppm)	NH_4 (ppm)	NO_3 (ppm)
Sandy	Sand	3.282	7.233	3.235	1644.667	70.521	9146.586	0.047	291.995
Clay+Sand	Loamy Sand	3.682	7.23	2.478	958.667	73.494	9140.756	0.052	315.509
Manure+Sand	Loamy Sand	7.952	6.933	1.669	829.333	76.094	9130.797	0.069	450.527
Compost+Sand	Loamy Sand	7.741	6.267	1.561	790.667	76.809	9079.943	0.071	381.501

Note: Each value was average of three sample of each treatment. ECe(2:5) stands for electrical conductivity of the saturation extract at a 2:5 Soil-to-water ratio. WHC stands for water holding capacity. Cl = chloride ions, NH_4= ammonium ions, NO_3 = nitrate ions.

Table S2. Water Quality parameters

Water quality	EC ($dS.m^{-1}$)	Total dissolved solids (ppm)	Nitrate ions (ppm)	Nitrite ions (ppm)	Chloride ions (ppm)	Fluoride ions (ppm)	Manganese ions (ppm)
Saline water	4.368	6240	0.4	0.002	2231.03	1.15	0.006
Fresh water	0.357	510	0.4	0.015	196.75	0.35	0.006

Note: EC = Electrical conductivity.

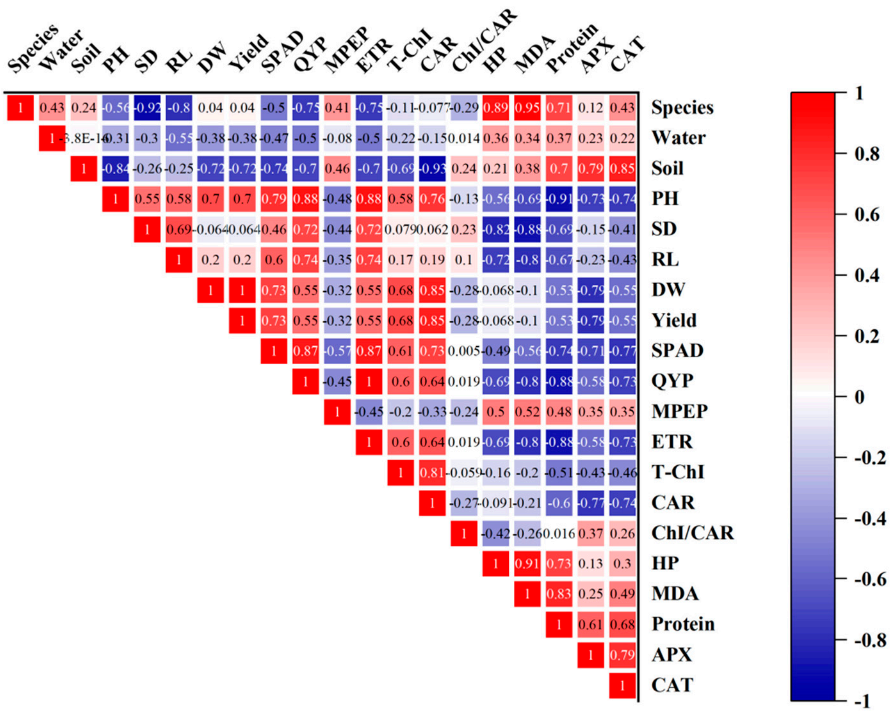

Figure S1. Correlation analysis between plant species, water treatment,soil amendment, growth traits, Chlorophyll parameters, photosynthesis pigments and enzyme activity. Red color indicate the negative correlation and blue color indicate the positive correlation. Note: Species = plant species, Water = water treatments, Soil = soil amendments, PH = plant height, SD = stem diameter, RL = root length, DW = dry weight per plant, SPAD = chlorophyll content, QYP = quantum yield of photosystem II, MPEP = maximal photochemical efficiency of photosystem II, ETR = electron transport rate, T-Chl = total chlorophyll, CAR = carotenoids, HP = hydrogen peroxide, MDA = malondialdehyde, APX = Ascorbate peroxidase, and CAT = Catalase.

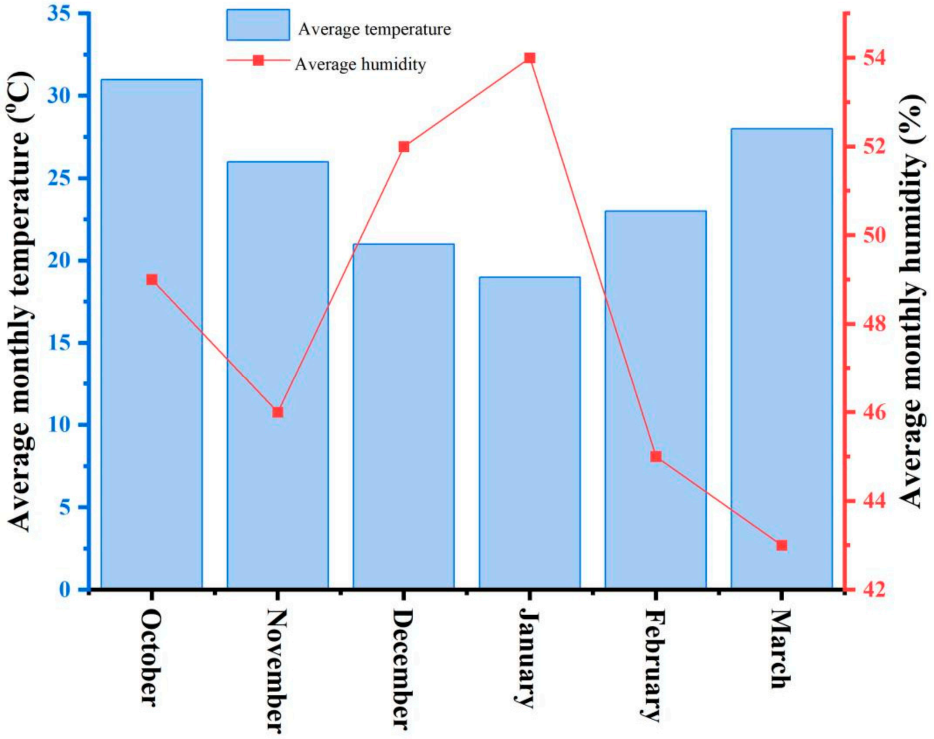

Figure S2. Average monthly temperature and humidity of the experimental period
